# The Uses of a Health Resort

**Published:** 1888-07-14

**Authors:** 


### The Doctor's Pulpit.

### THE USES OF A HEALTH RESORT.

Last week the choice of a health resort was discussed; this week the uses of such a resort may be fitly considered. A very modest expenditure on the annual holiday will reach towards fifty pounds. Such a sum, taken from an income of four or five hundred, is so considerable a proportion of the whole that it is of no slight importance to give some thought to its wise expenditure. A summer holiday to a hardworked town man is quite indispensable if he is to keep well: as indispensable as a new coat, a daily dinner, or any other positive necessity. Let the maximum health value it is possible to get out of such a holiday be taken at 100. Some men will get the whole 100 out of it, some only 90, some 50, some 20, and some will not get even 1; whilst a few will represent their health gains by a minus quantity, or in other words they will be worse at the end than at the beginning of their holiday.

What does a man hope to gain by leaving his town home and business, seeking a distant place of sojourn, and spending a sum of money which he can often ill afford? Either he hopes to gain some real and appreciable advantage or he is a very foolish fellow. Let us see what advantages may reasonably be expected. First of all there is a changed atmosphere to breathe and live in. The air of London and other large towns is not poisonous in any extreme degree. But no one will deny that it is much less perfect than absolutely pure air. To the extent to which it is less perfect than pure atmospheric air—to that extent it is poisonous and injurious to the health; to that extent it lowers the health standard, the pleasure standard, the efficiency standard, and the moral standard: to that extent it makes a man less full, less strong, less complete, and less effective in everything which constitutes a perfect and typical human being. The large town should therefore be periodically fled from, in order that the slowly poisonous and certainly injurious effects of its atmosphere may be checked and minimised as much as may be possible.

One of the prime conditions of a perfect health resort is an absolutely pure atmospheric air. That is why the sea or the mountain is so often sought. The sea offers little impediment to the free rush of air over its surface for thousand of miles. For thousands of miles there are no means of atmospheric contamination. The air on the shores of large ocean tracts is therefore usually perfect in its purity. Similarly, a mountain altitude of fifteen hundred or two thousand feet above the sea level is generally surrounded by an atmosphere high above the ordinary sources of impure admixture, and the air at such an altitude is often as pure as that of the sea. Of course, both sea and mountain air may have their own charactertistics, which it is sometimes necessary to take advantage of in certain classes of disease; but just now it is the question of atmospheric purity with which we are specially concerned. For the proper oxidation and purification of the blood, for the restoration and preservation of the normal condition of the air passages and air cells and their secreting structures, for the stimulus and healthy activity of the processes of waste, repair, and excretion in every part of the body, a perfectly pure air is always desirable, and at certain fixed periods it is absolutely indispensable, if a man's life is to be normally long and normally efficient.

New scenes and surroundings are hardly less important to a worked-out man or woman than pure air. A man's business may be, and often is, his prison. Let him but be overworked and underpaid for a sufficient length of time, and his daily surroundings will become almost intolerable to him. Everything reminds him of the iron necessity which cramps his freedom on all sides. Even his home may become to him but one of the scenes of his slavery. Many a man loses both health and heart by long tramping in the business or domestic treadmill. Some few find even their reason forsaking them under such circumstances. An early legend records of the Apostle John that he was one day amusing himself by playing with some tame doves. A hunter, fresh from the chase, expressed his astonishment that the venerable Apostle should condescend to such trifling. "Why is that bow in your hand, which has been bent all day, now unloosed from the string"? said the Apostle. "In order that by being unbent when it is not needed, it may retain its elasticity and strength," answered the hunter. "It is precisely for that reason that I, who have a life of strenuous labour, may the better do that work that I now and regularly seek some simple recreation like this." Such was the Apostle's wise reply. When a man or a woman has left business and home in pursuit of health, it is the highest wisdom to shut and lock the door of memory so securely that no part of the past can flow over into the present. "Live by the day" is a good rule always, but especially is it so when it is holiday time. The present and the present only, with its present sights, its present sounds, and its present experiences, should be cared for. Let the past be buried and the future shut out as completely as the mind can accomplish such oblivion.

New companionships and new thoughts may be sought and gained in the season of idleness and ease. Alas, for the Englishman! He seldom makes new friends. Still seldomer, perhaps, does he thoroughly possess his mind with new thoughts. In early life he plans out his purposes, and often pursues them with such exclusive devotion that there is no time either for the making of friends or the expansion and discipline of the mind. Whatever bears upon his practical schemes, that he will make himself master of; but anything beyond that, of scientfic or moral truth, of wide general outlook, of comparison and measurement, he takes no account of; and so he becomes a sorry mixture of strength and weakness, of practical sense and efficiency in one department, but of childish judgment and incapacity in a dozen others; a kind of a three-legged stool with one leg broken off which can never stand upright except when it is propped against a wall. A man who is ardent in the pursuit of his life's purposes, should, more than other men, strive to make use of his annual holiday, and of the opportunities of his health resort. Let such a man for the time throw away all his absorbing plans and schemes. Measured by any great standard, they are probably not of any very overwhelming significance. But whether great or small, he will be a thousand times more likely to carry them to a successful issue if he now and again banishes them entirely from his mind. Let him cease to be a mere propeller of business organisations, and become a man —if possible a boy—again. He knows little of the thoughts and purposes of his fellow-men, because of his too great absorption in his own. It will be of infinite service to him to enter into the minds of other men; to see things with their eyes, from their standpoint; and to measure things with their foot-rule. After all, "no man liveth to himself," or in himself, or by himself. The companionship of other men is invaluable, and the expansive power of new thoughts is indispensable if a man is to lead a continually progressive life to the end. Grooves and railway lines are fatal to the living soul. Freedom, space, deliverance from self-absorption are to be sought after by all means. A health resort may be used for the highest, or for the lowest, or for no purpose at all. He is a wise and wholesome man who uses it alone for the highest.

